# Neoadjuvant Treatment Including Targeted Therapy and Locoregional Interventions for Locally Advanced Thyroid Cancer: A Single-Center Retrospective Cohort Study

**DOI:** 10.3390/cancers18152406

**Published:** 2026-07-26

**Authors:** Tz-You Chen, Lay-San Lim, Yen-Hao Chen, Yi-Chia Chan, Shen-En Chou, Shun-Yu Chi, Yen-Hsiang Chang, Shun-Chen Huang, Wei-Che Lin, Chen-Kai Chou

**Affiliations:** 1 Department of Endocrinology and Metabolism, Kaohsiung Chang Gung Memorial Hospital, Chang Gung University College of Medicine, Kaohsiung 833, Taiwan; 2 Department of Hematology-Oncology, Kaohsiung Chang Gung Memorial Hospital, Chang Gung University College of Medicine, Kaohsiung 833, Taiwan; 3 Department of General Surgery, Kaohsiung Chang Gung Memorial Hospital, Chang Gung University College of Medicine, Kaohsiung 833, Taiwan; 4 Department of Nuclear Medicine, Kaohsiung Chang Gung Memorial Hospital, Chang Gung University College of Medicine, Kaohsiung 833, Taiwan; 5 Department of Pathology, Kaohsiung Chang Gung Memorial Hospital, Chang Gung University College of Medicine, Kaohsiung 833, Taiwan; 6 Department of Diagnostic Radiology, Kaohsiung Chang Gung Memorial Hospital, Chang Gung University College of Medicine, Kaohsiung 833, Taiwan

**Keywords:** thyroid cancer, neoadjuvant treatment, targeted therapy, radiofrequency ablation, transarterial embolization

## Abstract

**Background**: Locally advanced thyroid cancer is associated with a poor prognosis and high surgical morbidity. This study evaluated the feasibility and outcomes of neoadjuvant treatment including targeted therapy with locoregional interventions. **Methods**: We retrospectively analyzed 17 patients with locally advanced thyroid cancer who received neoadjuvant treatment, including tyrosine kinase inhibitors (TKIs), transarterial embolization (TAE), and radiofrequency ablation (RFA), either alone or in combination. The primary outcome was the proportion of patients who proceeded to R0 or R1 surgical resection following neoadjuvant treatment. Secondary outcomes included tumor response assessed using the Response Evaluation Criteria in Solid Tumors (RECIST), version 1.1, and changes in surgical morbidity evaluated using the MGH/MEEI–MSK–MD Anderson surgical morbidity complexity score (MMM score) and the Invasive Thyroid Class. **Results**: Five patients (29%) achieved R0/R1 resection, including four with R0 resection. Most patients demonstrated reduced tumor burden and surgical complexity, as reflected by improvements in the MMM score and the Invasive Thyroid Class. The objective response rate was 47% and the 12-month progression-free survival rate was 58.2%. **Conclusion**: Neoadjuvant treatment may improve tumor downstaging and surgical feasibility in selected patients with initially unresectable thyroid cancer. Further prospective studies are required to validate these findings.

## Simple Summary

Locally advanced thyroid cancers that cannot be safely removed by surgery are associated with poor outcomes and a high risk of complications. In recent years, targeted therapies and minimally invasive procedures have shown the potential for reducing tumor burden before surgery. This study evaluated the feasibility of neoadjuvant treatment—including targeted therapy, transarterial embolization, and radiofrequency ablation, used alone or in combination—in patients with locally advanced thyroid cancer. This individualized, multidisciplinary approach may improve tumor resectability and reduce surgical complexity in selected patients. These findings suggest that neoadjuvant treatment may be a potential option for select patients who are initially considered unsuitable for curative surgery.

## 1. Introduction

Thyroid cancer stands as the most prevalent endocrine malignancy worldwide [1,2]. With advances in diagnostic tools and growing public health awareness, the incidence of thyroid cancer has increased annually [3]. According to the latest statistics from Taiwan’s Ministry of Health and Welfare, thyroid cancer ranked 5th among cancers with the greatest increase in new cases by 2023 [4]. Although thyroid cancer typically has a good prognosis, some patients develop aggressive disease. For extensive tumors or those invading critical neck structures, aggressive surgical intervention may leave residual disease or significantly affect a patient’s quality of life. Therefore, there remains substantial therapeutic space for patients with advanced and/or unresectable thyroid cancer.

Neoadjuvant therapy is now widely used in locally advanced cancers, such as breast cancer, and provides benefits including tumor downstaging, organ preservation, and assessment of treatment response [5]. As our understanding of the molecular mechanisms of thyroid cancer deepens, multiple tyrosine kinase inhibitors (TKIs) have been approved for specific gene mutations for the metastatic or recurrent radioiodine-refractory differentiated thyroid cancer (RR-DTC) [6,7,8,9,10]. Although evidence supporting neoadjuvant therapy in locally advanced thyroid cancer remains limited, recent studies have demonstrated that neoadjuvant targeted therapy can significantly reduce tumor burden, thereby enabling patients who were initially considered inoperable to undergo R0/R1 surgical resection without compromising their quality of life [11,12,13,14,15].

With the evolution of minimally invasive techniques, radiofrequency ablation (RFA) and transarterial embolization (TAE) have emerged as potential therapeutic options for patients with thyroid disease. RFA has been increasingly used in the management of benign thyroid nodules and selected low-risk malignancies [16,17,18,19,20,21,22]. In contrast, TAE has been explored as an adjunctive or palliative approach to reduce tumor vascularity and alleviate compressive symptoms [23,24,25,26]. Although these modalities are not considered standard treatments for advanced thyroid cancer, they may play complementary roles in carefully selected cases. Consequently, this study aimed to evaluate the feasibility of surgical resection and the clinical outcomes in patients with locally advanced thyroid cancer undergoing neoadjuvant treatment, including systemic targeted therapy and locoregional interventions such as TAE and RFA.

## 2. Materials and Methods

### 2.1. Patient Selection and Study Design

**Figure 1:**
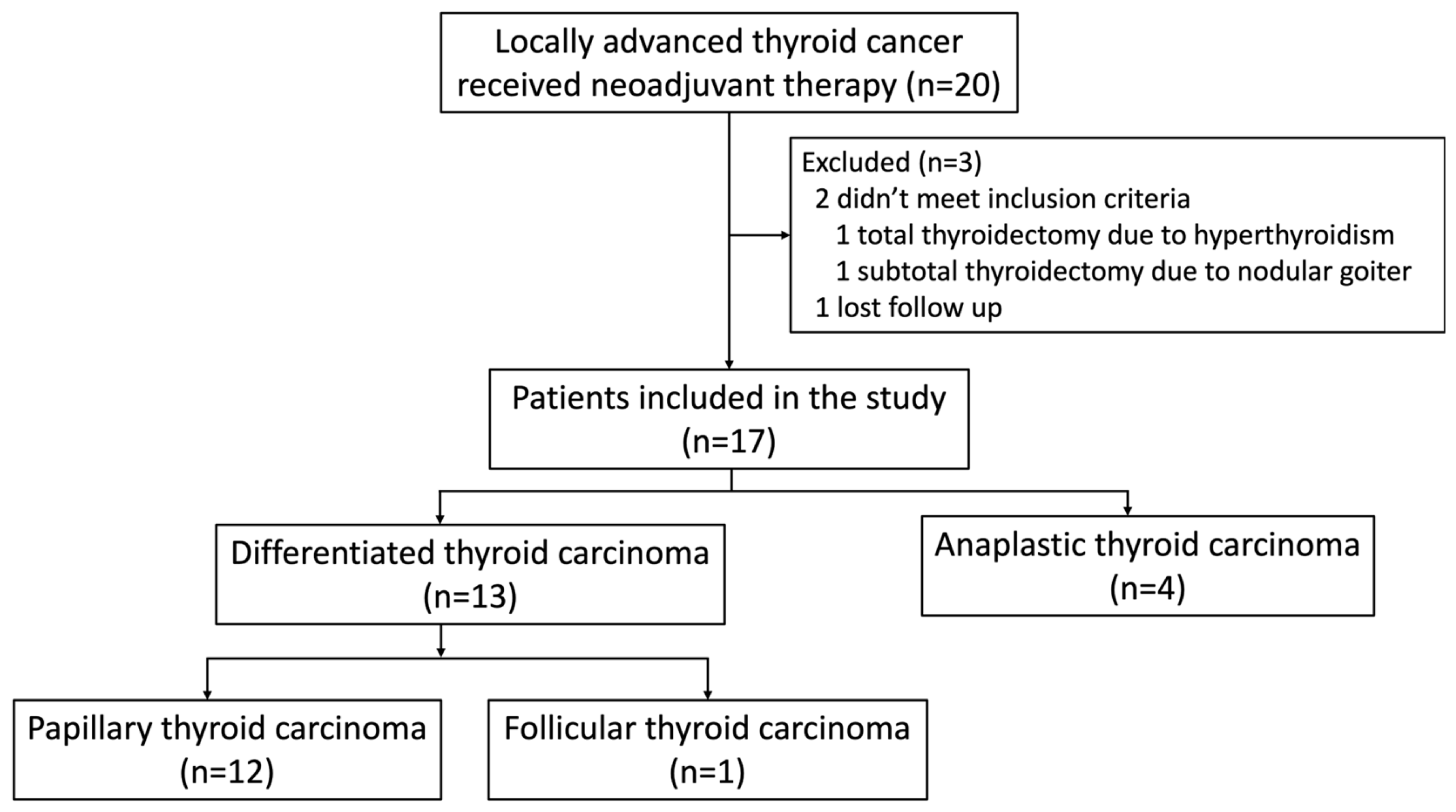
Flow diagram of patient selection.

This was a single-center retrospective cohort study. We reviewed the medical records of patients diagnosed with thyroid cancer, including differentiated thyroid cancer (DTC) and anaplastic thyroid cancer (ATC), at Kaohsiung Chang Gung Memorial Hospital between November 2018 and August 2025. All patients included in this study were evaluated by a multidisciplinary thyroid cancer team at our institution, consisting of endocrinologists, oncologists, surgeons, radiologists, nuclear medicine physicians, and pathologists. For the purposes of this study, locally advanced thyroid cancer was defined as T4a or T4b disease according to the 8th edition of the American Joint Committee on Cancer (AJCC) Staging System, with gross extrathyroidal extension involving critical cervical structures, including the larynx, trachea, esophagus, recurrent laryngeal nerve (RLN), prevertebral fascia, or major cervical arteries or veins, and deemed unlikely to achieve R0/R1 resection without major surgical morbidity based on preoperative cross-sectional imaging and multidisciplinary team assessment [27]. Neoadjuvant treatment was defined as preoperative strategies that included systemic targeted therapy with TKIs and locoregional interventions, specifically TAE and RFA. Patients were eligible if they met the following criteria: (1) age ≥ 18 years; (2) locally advanced thyroid cancer as defined above; and (3) receipt of at least one neoadjuvant treatment strategy, either alone or in combination, including TKI, TAE, or RFA. Patients with a history of thyroid surgery for goiter or hyperthyroidism were excluded. Seventeen patients met the eligibility criteria and were included in this study. The patient selection process is summarized in Figure 1.

To assess the extent of tumor invasion and determine the suitability of locoregional interventions, all patients underwent neck ultrasonography and cross-sectional imaging (computed tomography [CT] or magnetic resonance imaging [MRI]) before the initiation of treatment. Follow-up CT imaging was performed at 3-month intervals to evaluate treatment response. All TAE and RFA procedures were performed by experienced interventional radiologists at our institution.

### 2.2. Study Outcomes and Statistical Analysis

The primary outcome was the proportion of patients who proceeded to R0 or R1 surgical resection following neoadjuvant treatment. Secondary outcomes included changes in two surgical morbidity parameters—the MGH/MEEI–MSK–MD Anderson surgical morbidity complexity score (MMM score) and the Invasive Thyroid Class. These parameters were assessed according to the scoring system described by Russell et al., which was originally introduced in the protocol of the prospective phase II neoadjuvant lenvatinib trial (NCT04321954) [28,29]. Based on preoperative radiographic assessment of tumor involvement in critical anatomical structures, the scoring system was used to estimate surgical complexity and perioperative morbidity. Additional secondary outcomes included changes in tumor size assessed according to the Response Evaluation Criteria in Solid Tumors (RECIST), version 1.1.

Data collected included patient demographics, clinical and imaging findings, pathological features, tumor mutation status, and operative findings. Tumor staging was performed according to the AJCC 8th edition Staging System. Molecular alterations were assessed using immunohistochemical (IHC) staining. Continuous variables were expressed as mean ± standard deviation or median (interquartile range), as appropriate, while categorical variables were presented as frequencies and percentages. Comparisons between groups were performed using the Mann–Whitney U test for continuous variables and Fisher’s exact test for categorical variables. Statistical significance was set at *p* < 0.05. Statistical analyses were performed using SPSS Statistics (version 27.0; IBM, Armonk, NY, USA).

## 3. Results

### 3.1. Patient Characteristics

The baseline characteristics of the patients are summarized in Table 1. Seventeen patients were included in this analysis. The median age was 71 years (range, 24–85 years), and 9 patients (52.9%) were female. Most patients had a good performance status, with 14 (82.4%) having an Eastern Cooperative Oncology Group performance status of 0–1. Regarding tumor histology, 13 patients (76.5%) had DTC, including 12 patients (70.6%) with papillary thyroid carcinoma (PTC) and 1 patient (5.9%) with follicular thyroid carcinoma (FTC), while 4 patients (23.5%) had ATC. The median primary tumor diameter was 6.3 cm (range, 1.7–10.8 cm). Synchronous distant metastases were present in 9 patients (52.9%), most commonly involving the lung (29.4%), followed by the bone (17.6%), mediastinum (17.6%), and distant muscles (5.9%). Molecular alterations were identified in 10 patients (58.8%), including BRAF V600E mutations in 7 patients (41.2%), NRAS mutations in 2 (11.8%), and PIK3CA mutations in 1 (5.9%). The remaining 7 tumors showed no targetable alterations on IHC staining. Detailed baseline demographic and clinical features are provided in Appendix A Table A1.

The neoadjuvant treatment modalities and outcomes are summarized in Appendix A Table A2. Among the 13 patients with DTC (Cases 1–13), 7 received locoregional interventions (TAE and/or RFA) with or without TKI therapy, whereas the remaining 6 were treated with TKIs alone. All 4 patients with ATC (Cases 14–17) received targeted therapy alone. Neoadjuvant TKI regimens included lenvatinib, sorafenib, dabrafenib plus trametinib, and alpelisib plus trametinib. The duration of TKI treatment varied considerably among the patients. Among the 15 patients who received TKI therapy, the median duration of treatment was 5 months (range, <1–41 months), calculated as the cumulative duration of all sequential TKI regimens received.

### 3.2. Changes in Surgical Morbidity and Resectability After Neoadjuvant Treatment

**Figure 2:**
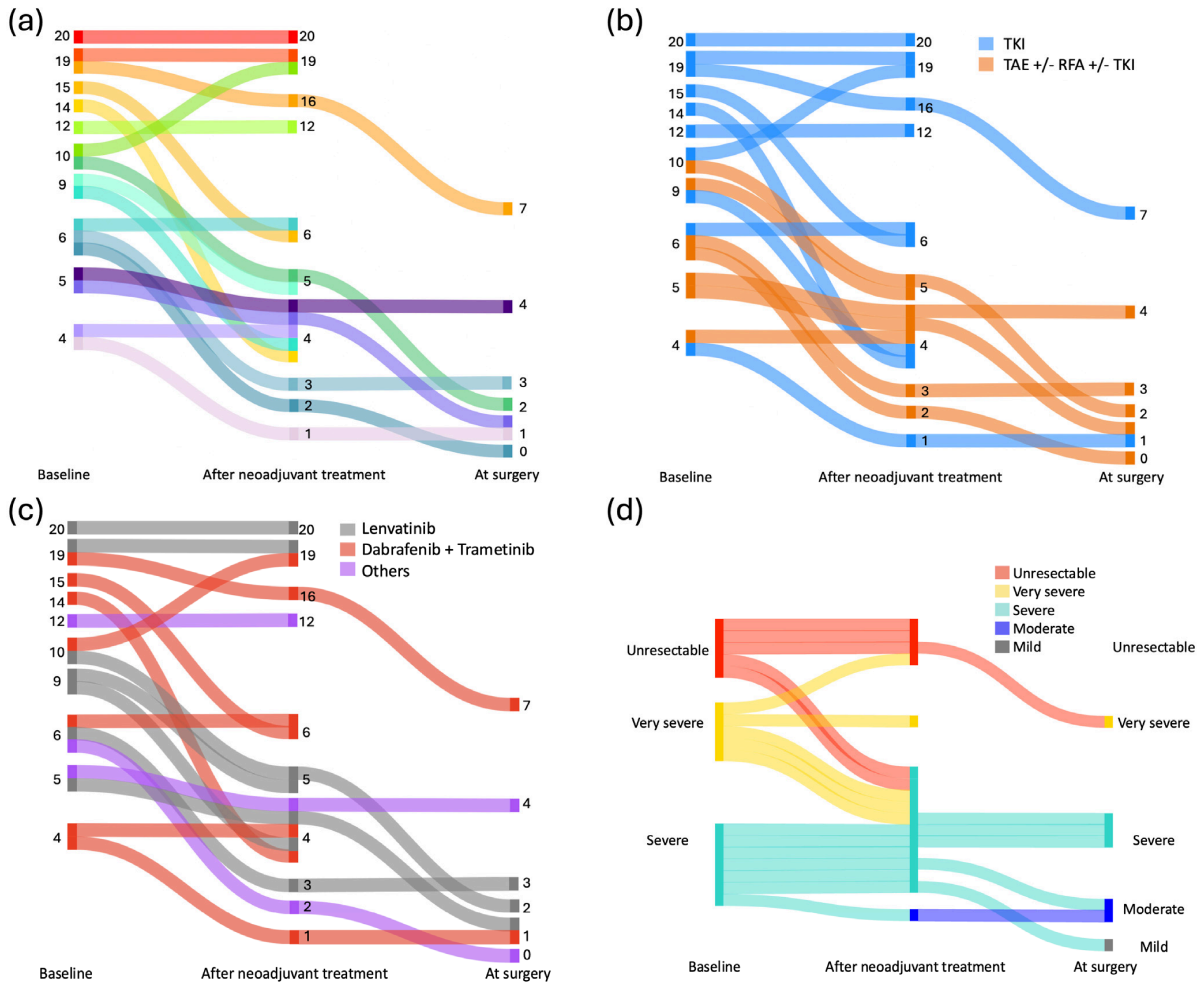
Sankey diagrams illustrating changes in MMM scores and Invasive Thyroid Class following neoadjuvant treatment in patients with locally advanced thyroid cancer. (**a**) Changes in MMM scores from baseline, after neoadjuvant treatment, and at surgery. Each colored flow represents an individual patient. (**b**) Changes in MMM scores according to treatment modality (TKI alone vs. locoregional interventions ± TKI). (**c**) Changes in MMM scores stratified by initial TKI regimen. (**d**) Distribution of Invasive Thyroid Class across treatment stages.

At baseline, all tumors were considered to carry a high risk of surgical morbidity. For the primary endpoint, 5 of the 17 patients (29%) achieved R0 or R1 resection after neoadjuvant treatment, including 4 with R0 resection and 1 with R1 resection. In addition, 2 patients underwent R2 resection. For the secondary endpoints, the mean MMM scores at baseline, after neoadjuvant treatment, and at the time of surgery were 10.1 ± 5.4, 7.8 ± 6.4, and 2.6 ± 2.4, respectively. According to the Invasive Thyroid Class, 5 of the 17 patients (29%) were classified as unresectable at baseline, 5 (29%) as very severe, and 7 (41%) as severe. After neoadjuvant treatment, 4 (24%) remained unresectable; 1 (6%) very severe; 11 (65%) severe; and 1 (6%) moderate. Among the 7 patients who underwent surgery, disease severity was further reduced, with 1 (14%) classified as very severe, 3 (43%) as severe, 2 (29%) as moderate, and 1 (14%) as mild. Baseline and post-neoadjuvant treatment MMM scores and Invasive Thyroid Class were assessed based on imaging findings, whereas surgical MMM scores and Invasive Thyroid Class were determined according to intraoperative findings at the time of surgery. The changes in each patient’s MMM scores and Invasive Thyroid Class are shown in Figure 2.

**Figure 3:**
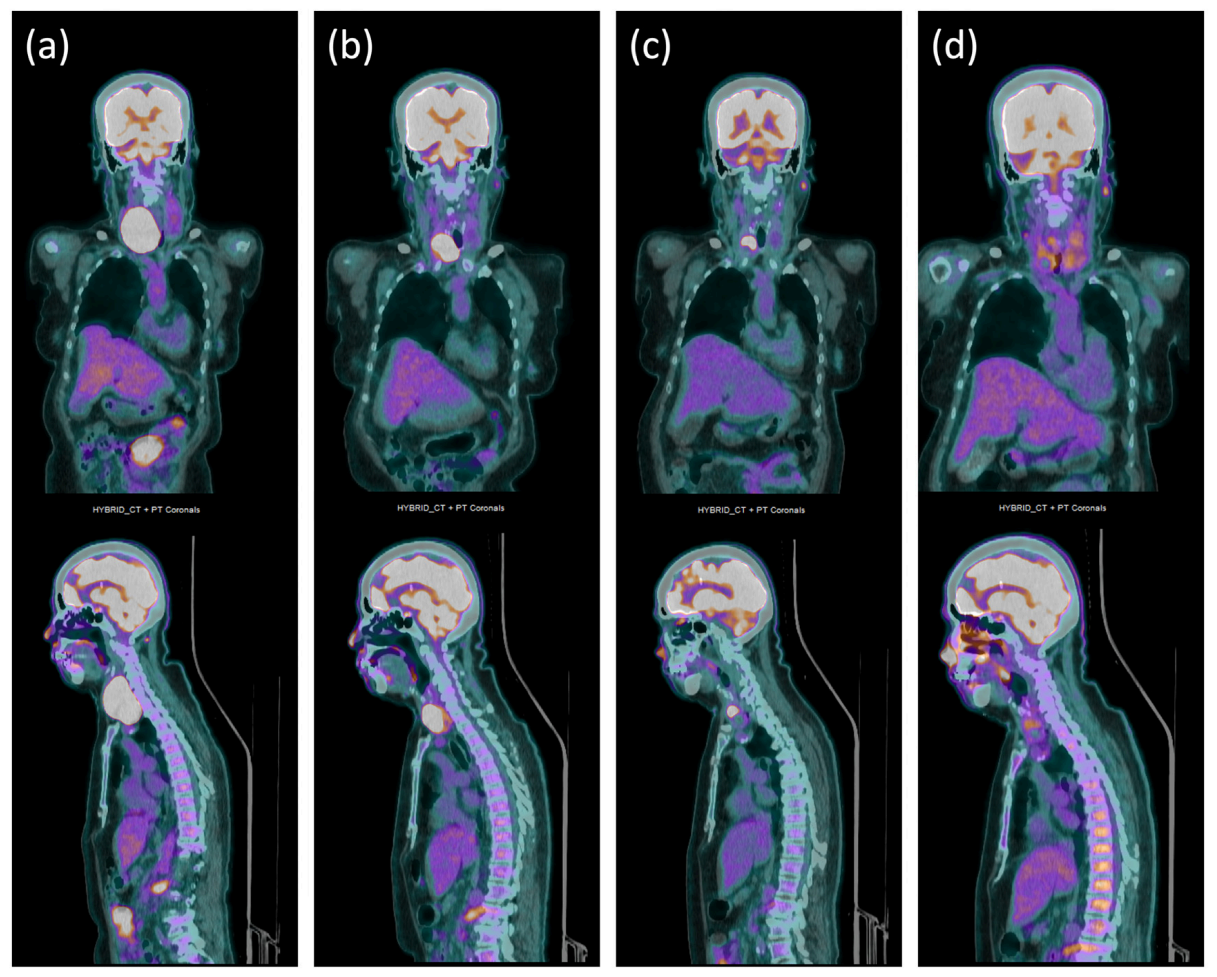
Serial PET/CT scans of Case 4 demonstrating progressive tumor regression following locoregional interventions: (**a**) baseline image showing a large hypermetabolic right thyroid mass with tracheal compression and deviation; (**b**) 4 months after TAE; (**c**) 12 months after TAE and 7 months after the first session of RFA; (**d**) 20 months after TAE, 15 months after the first session of RFA and 6 months after the second session of RFA.

Among our 17 patients, two (Cases 3 and 4) were unique in that they underwent locoregional intervention alone, consisting of TAE and RFA, without systemic targeted therapy. For example, Case 4 was a 71-year-old woman with locally advanced PTC who presented with a large right thyroid mass measuring 7.3 cm, involving the trachea and RLN, with associated tracheal compression and deviation. She underwent individualized neoadjuvant treatment, including TAE followed by two sessions of RFA, resulting in marked tumor regression on serial imaging. The patient subsequently underwent total thyroidectomy and achieved R0 resection, with no residual malignancy identified on final pathology. Serial PET/CT scans of Case 4 are shown in Figure 3. In contrast, three patients (Cases 8, 16, and 17) experienced early mortality within one month despite therapy, reflecting the aggressive nature of the disease and the limited opportunity for reassessment after treatment. Consequently, both the MMM score and the Invasive Thyroid Class remained unchanged in these patients. One patient (Case 12) with a BRAF V600E mutation received sequential TKI therapy and was initially treated with dabrafenib plus trametinib for 17 months, followed by lenvatinib because of disease progression, and subsequently sorafenib after the development of new metastatic lesions. The patient ultimately died from massive bleeding secondary to carotid artery blowout. Following review by our multidisciplinary thyroid cancer team, this event was attributed to direct tumor invasion and progressive encasement of the common carotid artery by the locally advanced PTC, rather than to treatment-related toxicity. Apart from these four patients (Cases 8, 12, 16, and 17), most patients demonstrated stable or improved local disease status, as reflected by decreased MMM scores and lower Invasive Thyroid Class following neoadjuvant treatment.

At the data cutoff, 2 (12%) patients achieved complete response (CR) and 6 (35%) achieved partial response (PR), yielding an objective response rate (ORR) of 47%. An additional 4 patients (24%) had stable disease (SD), while 1 (6%) had progressive disease (PD). The mortality rate was 24% (4 of 17 patients). The median follow-up duration was 19 months (range, 1–69). The 12-month progression-free survival (PFS) rate was 58.2%; 3 patients had not yet reached 12 months of follow-up at the time of analysis. Excluding 3 patients who died within 1 month of treatment initiation (Cases 8, 16, and 17), the remaining 14 patients showed a median reduction of 22.6% in the largest primary tumor diameter.

### 3.3. Comparison of Baseline Characteristics by Treatment Modality

Comparisons of the baseline characteristics between patients who underwent surgery and those who did not are summarized in Table 2. Overall, the two groups were generally comparable at baseline, with no significant differences observed in age, sex distribution, body mass index (BMI), primary tumor size, initial MMM score, initial Invasive Thyroid Class, differentiated thyroid cancer histology, BRAF V600E mutation status, or the presence of distant metastasis. The baseline characteristics according to treatment modality are presented in Table 3. The locoregional intervention group (TAE and/or RFA) and the TKI alone group were generally comparable in terms of age, sex distribution, BMI, primary tumor size, histological subtype, BRAF V600E mutation status, and the presence of distant metastasis. However, patients who underwent locoregional intervention had significantly lower initial MMM scores compared with those treated with TKI alone (median 6.0 [IQR, 5.0–9.0] vs. 13.0 [IQR, 8.3–18.3], *p* = 0.021). Similarly, the initial Invasive Thyroid Class was significantly lower in the locoregional intervention group (median 2.0 [IQR, 2.0–3.0] vs. 3.5 [IQR, 2.8–4.0], *p* = 0.017). Although not statistically significant, the locoregional intervention group also showed a numerically higher rate of conversion to R0/R1 resection than the TKI-only group (57.1% vs. 10.0%, *p* = 0.101). However, given the significantly lower baseline disease severity in the locoregional intervention group, direct comparison of surgical conversion rates between groups should be interpreted with caution.

### 3.4. Safety Outcomes

Adverse events related to neoadjuvant treatment were generally mild and well tolerated, with most TKI-related toxicities being grade 1–2 in severity. The reported toxicities included proteinuria, hypertension, hand–foot syndrome, anemia, fatigue, and fever. No patients required permanent discontinuation of TKI therapy due to adverse events. Regarding locoregional interventions, only one patient developed transient swallowing pain following the procedure. No treatment-related mortality was observed.

## 4. Discussion

### 4.1. Principal Findings and Clinical Implications

This is the first study to incorporate locoregional interventions, including TAE and RFA, into the neoadjuvant treatment strategy for locally advanced thyroid cancer. In this real-world cohort, neoadjuvant treatment enabled a subset of patients with initially high surgical morbidity to undergo definitive surgery with reduced tumor burden and structural invasion, allowing for less extensive resection and preservation of critical organ function. Notably, 5 of the 17 patients (29%) achieved R0/R1 resection, and most demonstrated reductions in surgical complexity, as reflected by improvements in the MMM score and Invasive Thyroid Class. Enhanced local disease control may facilitate the resection of the primary lesion and allow for postoperative RAI therapy, even in patients with lymph node or distant metastases. Importantly, not all patients who did not proceed to surgery should be considered treatment failures. In our cohort, some patients elected to defer surgical intervention after achieving disease stabilization, reflecting patient preference to avoid the risks of surgery. These findings underscore that resectability represents a clinically nuanced endpoint rather than a simple binary outcome.

Regarding the differential use of treatment modalities, patients who underwent locoregional intervention (TAE and/or RFA) demonstrated significantly lower baseline MMM scores and Invasive Thyroid Class compared with those treated with TKI alone. This likely reflects real-world treatment selection based on procedural feasibility rather than disease severity alone. Locoregional interventions are more likely to be considered in patients with anatomically localized disease, preserved procedural accessibility, and a lower risk of catastrophic complications, such as carotid blowout or extensive aerodigestive invasion. Conversely, patients with BRAF V600E mutations, distant metastases, highly infiltrative tumors, diffuse vascular encasement, or rapidly progressive disease were more commonly managed with systemic therapy. Given this baseline imbalance, the observed difference in surgical conversion rates (57.1% vs. 10.0%) should not be interpreted as evidence of superior efficacy of locoregional intervention over systemic therapy; instead, it likely reflects inherent selection bias in this retrospective cohort. Prospective studies with pre-specified, standardized eligibility criteria are needed to properly evaluate the independent contribution of each treatment modality to surgical conversion.

### 4.2. Current Evidence on Neoadjuvant Targeted Therapy and Locoregional Interventions

Neoadjuvant targeted therapy has been incorporated into current guidelines for selected patients with ATC [30], particularly those harboring actionable mutations. However, the role of DTCs in the neoadjuvant setting remains less well established. Recent studies have demonstrated the feasibility of neoadjuvant targeted therapy in locally advanced thyroid cancer. In a prospective phase II trial of 13 patients with locally advanced DTC, neoadjuvant anlotinib achieved an R0/R1 resection rate of 61.5% [11]. In another prospective cohort of 14 patients with locally advanced DTC treated with neoadjuvant apatinib, 92.9% of patients were able to proceed to surgical resection following therapy [13]. Real-world evidence supports the use of this approach. In a cohort of 27 patients with advanced thyroid cancer treated with neoadjuvant TKI therapy, including 18 with DTC, 6 with medullary thyroid cancer, and 3 with ATC, R0/R1 resection was achieved in 6 patients with DTC (33.3%) and 1 patient with ATC (33.3%) [14]. In another cohort of 17 patients, 10 patients (58.8%) achieved R0/R1 resection following neoadjuvant therapy. Surgical complexity, as measured by the MMM score and Invasive Thyroid Class, decreased consistently across treatment time points [28]. Furthermore, a systematic review including 119 patients across multiple studies reported tumor volume reductions ranging from 25% to 87%. Among the 114 patients included in the conversion surgery analysis, 83 (72.8%) achieved R0/R1 resection [31]. Taken together, these findings support the emerging role of neoadjuvant targeted therapy as a strategy to downstage disease and improve surgical feasibility in locally advanced thyroid cancer.

In parallel with the evolving role of systemic therapy, locoregional treatment strategies have also gained increasing attention in thyroid cancer management. In recent years, RFA has been formally incorporated into multiple international guidelines as a treatment option for selected patients with low-risk PTC (cT1aN0M0), particularly for those who are not ideal surgical candidates or who prefer minimally invasive approaches [20,21,32]. Beyond its established role in low-risk disease, several studies have suggested that TAE and RFA may serve as adjunctive modalities in advanced thyroid cancer, including recurrent and selected locally advanced disease, traditionally with palliative intent. In a cohort of 20 patients with thyroid tumors, including 7 cases of inoperable ATC and 13 cases of DTC, selective embolization of thyroid arteries effectively reduced tumor vascularity, induced ischemic necrosis, and led to symptomatic improvement [25]. Moreover, a systematic review demonstrated that TAE has been applied to various thyroid conditions, including goiters, Graves’ disease, and thyroid malignancies, where it alleviated symptoms and facilitated safe surgical resection in selected cases [26]. Notably, in our cohort, one patient (Case 4) underwent TAE followed by two sessions of RFA and subsequently achieved R0 resection, with no residual malignancy identified on final pathology. These findings suggest that locoregional therapies for locally advanced thyroid cancer are no longer limited to purely palliative or adjunctive applications, but they may represent a potentially curative approach in carefully selected patients.

### 4.3. Study Limitations and Future Perspectives

This study has several limitations. First, its retrospective design, small sample size, and single-center setting may limit the generalizability of the findings and introduce potential selection bias. Second, the heterogeneity of tumor characteristics and treatment strategies, including variations in TKI regimens, treatment duration, and the use of locoregional interventions, may confound the interpretation of treatment effects. The wide range of treatment durations (from less than one month to 41 months) reflects both the individualized nature of multidisciplinary decision-making and the absence of established guidelines for optimal preoperative TKI duration in this setting. In cases with prolonged treatment courses, the boundary between neoadjuvant intent and ongoing disease control becomes inherently blurred. Finally, the relatively short follow-up duration in some patients limited the evaluation of long-term oncologic outcomes. Further large-scale prospective studies are warranted to validate these findings, establish robust criteria for optimal patient selection, and better define the role of these strategies in clinical practice.

## 5. Conclusions

Surgical resection followed by RAI therapy remains the standard of care for thyroid cancer. Our study highlights the potential role of combining targeted therapy with minimally invasive approaches, such as TAE and RFA, as neoadjuvant treatment strategies for patients with locally advanced thyroid cancer initially considered unresectable or associated with high surgical morbidity. However, treatment decisions should be individualized, carefully balancing potential therapeutic benefits against treatment-related risks while ensuring active patient participation in the decision-making process. Overall, our findings provide real-world evidence supporting the potential of neoadjuvant treatment to improve surgical resectability in selected patients with locally advanced thyroid cancer.

## Figures and Tables

**Table 1:** Baseline clinical and tumor characteristics of patients with locally advanced thyroid cancer.

Characteristic	*n*	%
Age, median (range), years	71(24–85)	
Sex		
Male	8	47.1
Female	9	52.9
ECOG		
0	6	35.3
1	8	47.1
2	3	17.6
Thyroid cancer histotype		
Differentiated thyroid cancer	13	76.5
Papillary thyroid carcinoma	12	70.6
Follicular thyroid carcinoma	1	5.9
Anaplastic thyroid cancer	4	23.5
Primary tumor diameter, median (range), cm	6.3 (1.7–10.8)	
Distant metastasis	9	52.9
Lung	5	29.4
Bone	3	17.6
Mediastinum	3	17.6
Distant muscles	1	5.9
Molecular mutations	10	58.8
BRAF V600E	7	41.2
NRAS	2	11.8
PIK3CA	1	5.9

Abbreviations: ECOG, Eastern Cooperative Oncology Group performance status.

**Table 2:** Comparison of baseline characteristics between the surgery and non-surgery groups.

Variable	Surgery (*n* = 7)	Non-Surgery (*n* = 10)	*p* Value
Age, years	67.0 (IQR: 63.0–74.0)	75.0 (IQR: 64.5–78.0)	0.350
Male sex	3 (42.9%)	5 (50.0%)	0.335
BMI, kg/m^2^	23.7 (IQR: 22.2–23.9)	25.6 (IQR: 20.9–28.0)	0.380
Primary tumor size, cm	5.7 (IQR: 5.3–7.3)	7.6 (IQR: 4.1–8.5)	0.700
Initial MMM score	6.0 (IQR: 5.0–10.0)	11.0 (IQR: 8.3–15.8)	0.120
Initial Invasive Thyroid Class	2.0 (IQR: 2.0–3.0)	3.0 (IQR: 2.8–4.0)	0.068
Differentiated thyroid cancer	6 (85.7%)	7 (70.0%)	0.603
BRAF V600E mutation	2 (28.6%)	5 (50.0%)	0.622
Distant metastasis	2 (28.6%)	7 (70.0%)	0.153

Abbreviations: BMI, body mass index; IQR, interquartile range; MMM score, MGH/MEEI–MSK–MD Anderson surgical morbidity score. Invasive Thyroid Class was numerically coded as follows: mild = 0, moderate = 1, severe = 2, very severe = 3, and unresectable = 4.

**Table 3:** Comparison of baseline characteristics and surgical outcomes between the locoregional intervention group and TKI alone group.

Variable	Locoregional Intervention, TAE and/or RFA (*n* = 7)	TKI Alone (*n* = 10)	*p* Value
Age, years	71.0 (IQR: 63.0–77.0)	70.5 (IQR: 64.5–77.3)	0.695
Male sex	3 (42.9%)	5 (50.0%)	1.000
BMI, kg/m^2^	23.9 (IQR: 23.7–27.8)	22.6 (IQR: 20.9–27.1)	0.329
Primary tumor size, cm	5.3 (IQR: 3.9–7.3)	7.6 (IQR: 4.8–8.6)	0.283
Initial MMM score	6.0 (IQR: 5.0–9.0)	13.0 (IQR: 8.3–18.3)	0.021
Initial Invasive Thyroid Class	2.0 (IQR: 2.0–3.0)	3.5 (IQR: 2.8–4.0)	0.017
Differentiated thyroid cancer	7 (100.0%)	6 (60.0%)	0.103
BRAF V600E mutation	1 (14.3%)	6 (60.0%)	0.134
Distant metastasis	2 (28.6%)	7 (70.0%)	0.153
Conversion to R0/R1 resection	4 (57.1%)	1 (10.0%)	0.101

Abbreviations: TAE, transarterial embolization; RFA, radiofrequency ablation; TKI, tyrosine kinase inhibitor. Invasive Thyroid Class was numerically coded as follows: mild = 0, moderate = 1, severe = 2, very severe = 3, and unresectable = 4.

## Data Availability

The data supporting the findings of this study are not publicly available due to privacy and ethical restrictions.

## Abbreviations

The following abbreviations are used in this manuscript:

AJCC	American Joint Committee on Cancer
ATC	Anaplastic thyroid cancer
DTC	Differentiated thyroid cancer
FTC	Follicular thyroid carcinoma
PTC	Papillary thyroid carcinoma
RR-DTC	Radioiodine-refractory differentiated thyroid cancer
TKI	Tyrosine kinase inhibitor
TAE	Transarterial embolization
RFA	Radiofrequency ablation
RAI	Radioactive iodine
MMM	MGH/MEEI–MSK–MD Anderson surgical morbidity score
RECIST	Response Evaluation Criteria in Solid Tumors
CR	Complete response
PR	Partial response
SD	Stable disease
PD	Progressive disease
ECOG	Eastern Cooperative Oncology Group
BMI	Body mass index
IHC	Immunohistochemical
IQR	Interquartile range
ORR	Objective response rate
PFS	Progression-free survival
RLN	Recurrent laryngeal nerve

## Appendix A

**Table A1:** Baseline clinicopathological characteristics of patients with locally advanced thyroid cancer.

Case	Age (years)	Sex	Histology	Molecular Mutations	Stage	Metastatic Site	Primary Tumor (cm)
1	77	M	FTC	NRAS Q61R	T4aN1bM1	lung	5.3
2	74	F	PTC	NRAS Q61R	T4aN1bM1	mediastinum	5.3
3	24	F	PTC	-	T4aN1bM0	-	6.3
4	71	F	PTC	-	T4aN0bM0	-	7.3
5	63	M	PTC	-	T4aN1aM0	-	1.7
6	77	M	PTC	-	T4aN1bM0	-	8.7
7	66	F	PTC	BRAF V600E	T4aN1bM0	-	3.9
8	65	M	PTC	BRAF V600E	T4bN1bM1	lung, bone, distant muscles	7.0
9	85	F	PTC	BRAF V600E	T4aN1bM1	lung	3.7
10	67	F	PTC	BRAF V600E	T4bN1bM0	-	10.8
11	76	M	PTC	BRAF V600E	T4bN1bM1	bone	8.2
12	76	M	PTC	BRAF V600E	T4bN1aM1	lung, bone	4.2
13	56	M	PTC	-	T4bN1bM0	-	8.4
14	67	F	ATC	BRAF V600E	T4aN1bM0	-	5.7
15	74	F	ATC	-	T4aN1bM1	mediastinum	5.0
16	81	F	ATC	-	T4aN1bM1	lung	8.1
17	63	M	ATC	PIK3CA	T4bN1bM1	mediastinum	9.2

Abbreviations: FTC, follicular thyroid carcinoma; PTC, papillary thyroid carcinoma; ATC, anaplastic thyroid carcinoma.

**Table A2:** Neoadjuvant treatment modalities and clinical outcomes of patients with locally advanced thyroid cancer.

Case	TAE	RFA	TKI (Duration, Months)	MMM Score			Invasive Thyroid Class			Resection Status	Status at Last Follow-Up
				Baseline	After Neoadjuvant	OP	Baseline	After Neoadjuvant	OP		
1	Y	Y	L (5)	6	3	3	Severe	Severe	Severe	R1	PR
2	Y	Y	L (4)	5	4	1	Severe	Severe	Moderate	R0	CR
3	Y	Y	-	5	4	4	Severe	Severe	Severe	R2	SD
4	Y	Y	-	6	2	0	Severe	Severe	Mild	R0	CR
5	Y	-	L (17)	10	5	2	Very severe	Severe	Severe	R0	PR
6	Y	-	L (1)	9	5	-	Very severe	Severe	-	-	PR
7	Y	-	D + T (4)	4	4	-	Severe	Severe	-	-	PR
8	-	-	D + T (<1)	6	6	-	Severe	Severe	-	-	PD (death)
9	-	-	D + T (41)	14	4	-	Unresectable	Severe	-	-	SD
10	-	-	D + T (7)	19	16	7	Unresectable	Unresectable	Very severe	R2	PR
11	-	-	D + T (4) −> L (31)	15	6	-	Unresectable	Severe	-	-	SD
12	-	-	D + T (17) −> L (6) −> Sor (1)	10	19	-	Very severe	Unresectable	-	-	PD (death)
13	-	-	L (39)	20	20	-	Unresectable	Unresectable	-	-	PD
14	-	-	D + T (5)	4	1	1	Severe	Moderate	Moderate	R0	SD
15	-	-	L (12)	9	4	-	Very severe	Severe	-	-	PR
16	-	-	L (<1)	19	19	-	Unresectable	Unresectable	-	-	PD (death)
17	-	-	A + T (<1)	12	12	-	Very severe	Very severe	-	-	PD (death)

Abbreviations: TAE, transarterial embolization; RFA, radiofrequency ablation; TKI, tyrosine kinase inhibitor; MMM, MGH/MEEI–MSK–MD Anderson surgical morbidity score; OP, operation; PR, partial response; CR, complete response; SD, stable disease; PD, progressive disease; L, lenvatinib; D + T, dabrafenib plus trametinib; Sor, sorafenib; A + T, alpelisib plus trametinib; Y indicates treatment performed; “-” indicates treatment not performed.
